# A Seven Immune-Related lncRNAs Model to Increase the Predicted Value of Lung Adenocarcinoma

**DOI:** 10.3389/fonc.2020.560779

**Published:** 2020-10-14

**Authors:** Jian-Ping Li, Rui Li, Xiao Liu, Chen Huo, Ting-Ting Liu, Jie Yao, Yi-Qing Qu

**Affiliations:** ^1^Department of Pulmonary and Critical Care Medicine, Qilu Hospital, Cheeloo College of Medicine, Shandong University, Jinan, China; ^2^Department of Pulmonary and Critical Care Medicine, Qilu Hospital of Shandong University, Jinan, China

**Keywords:** lung adenocarcinoma, immune-related lncRNAs, GSEA analysis, prognosis, predicted model

## Abstract

**Background:**

Recent research has shown that immune-related lncRNA plays a crucial part in the tumor immune microenvironment. This study tried to identify immune-related lncRNAs and construct a robust prediction model to increase the predicted value of lung adenocarcinoma (LUAD).

**Methods:**

RNA expression data of LUAD were download from the Cancer Genome Atlas (TCGA) database. Immune genes were acquired from the Molecular Signatures Database (MSigDB). The immune gene related lncRNAs were acquired by the “limma R” package and Cytoscape3.7.1. Cox regression analysis was applied to construct this forecast model. The prognostic model was validated by the testing cohort which was acquired by the bootstrap method.

**Results:**

A total of 551 lncRNA expression profiles including 497 LUAD tissues and 54 non-LUAD tissues were obtained. A total of 331 immune genes were acquired. The result of the Cox regression analysis showed that seven lncRNAs (AC022784-1, NKILA, AC026355-1, AC068338-3, LINC01843, SYNPR-AS1, and AC123595-1) can be performed to construct the prediction model to forecast the prognosis of LUAD. Kaplan–Meier curves indicated that our prediction model can distribute LUAD patients into two different risk groups (high and low) with significant statistical significance (*P* = 1.484e-07). Cox analysis and independent analysis illustrated that the seven-lncRNAs prediction model was an isolated factor by comparing it with other clinical variables. We validated the accuracy of our model in the testing dataset. Furthermore, the prognostic model also showed higher predictive efficiency than three other published prognostic models. The two different survival groups represented diverse immune features according to principal components analysis. GSEA analysis (gene set enrichment analysis) indicated that seven-lncRNAs signatures may be involved in the progression of tumorigenesis.

**Conclusions:**

We have established a seven immune-related lncRNAs prediction model. This prognostic model had significant clinical significance that increased the predicted value and guided the personalized treatment for LUAD patients.

## Introduction

Lung cancer belongs to the malignant tumor group and has becoming the primary killer in tumor-related disease (1, 2). Lung cancer is separated into two important categories including small cell lung cancer (SCLC) and non-small cell lung cancer (NSCLC) (3, 4). Lung adenocarcinoma (LUAD) is the major type of NSCLC and the morbidity of LUAD has surpassed lung squamous carcinoma in recent years (5). Due to the deficiency of tumor diagnosis using the traditional bronchoscopy and computed tomography techniques, patients in the early stage of cancer are difficult to be detected (6, 7). Therefore, it is imperative to find prognostic biomarkers that instruct the treatment of lung cancer. Currently, a lot of attention has been given to the field of immune therapies that could regulate the tumor microenvironment (8–10). For example, a study shows the relationship between the lncRNA signature of tumor-infiltrating B lymphocytes and the immune therapies of bladder cancer (11). Additionally, lncRNAs have several significant biological functions. For example, they participate in high-order chromosomal dynamics and mediate epigenetic changes (12, 13). Furthermore, lncRNA has an important value in evaluating the immune infiltrate of the tumor (14). Recently, Song et al. found that a gene signature including 30 immune-related genes could predict prognosis and reveal the relationship between the tumor and the immune microenvironment (15). Li et al. found that the clinical immune signature can act as a conspicuous marker to evaluate the overall survival rate in NSCLC and patients in the early phase (16). Prognostic biomarkers based on the immune-related lncRNA model for LUAD is still lacking.

In this study, our efforts concentrated on obtaining lncRNA expression profiles and immune genes to establish a prediction model to enhance the prognosis ability of LUAD. Then we identified whether our prognostic model was connected with the survival time of LUAD patients and independent of other clinical variables. Finally, we aimed to show the possible biological pathway of the prediction model.

## Materials and Methods

### Publicly Attainable Expression Datasets

The RNA-seq FPKM (reads per kilobase per million) data of 551 LUAD patients (including 497 LUAD tissues and 54 non-LUAD tissues) were download from The Cancer Genome Atlas (TCGA) database.^1^ Non-LUAD tissue refers to adjacent normal material. There were 445 LUAD patients, including their clinical follow-up information, who were taken into consideration. We chose patients whose survival data >30 days in order to improve the accuracy of our study (17). Patients whose survival data <30 days may die of other diseases rather than LUAD.

### Immune-Related lncRNAs

The immune genes were download from the Molecular Signatures Database (MSigDB).^2^ The “limma R” package was used to detect immune lncRNAs. The immune-related lncRNAs were identified by the correlation analysis between the immune genes and lncRNA expression levels in the LUAD samples. We used the function of cor. test () to calculate the correlation coefficient. We set the coefficient of the cor-Filter >0.6 and *P* value < 0.001.

### Prognosis Model Development

Cox regression analysis was used to build a prognosis signature of survival using the “survival R” package. lncRNAs with prominently statistical significance in univariable Cox regression were chosen and put into multivariable Cox regression. The risk score of every LUAD patient was computed based on the expression quantity of the model lncRNAs and their coefficient. The risk score was calculated as: risk score = β_gene__1_ × Expression_gene__1_ + β_gene__2_ × Expression_gene__2_ + β_gene__3_ × Expression_gene__3_ + … + β_genen_ × Expression_genen_ (18). Then we divided the LUAD patients into high and low risk groups according to the median risk score.

### Prognostic and Independent Analysis

A Kaplan–Meier survival curve were performed to identify the difference of overall survival in the two different risk groups in the training set. We used the “survival R” package and “survminer R” package to make the K–M survival curve. Then independent analysis was applied to verify the independence of our prediction model by comparing age, gender, stage, and TNM (pathological T stage, pathological N stage, and pathological M stage) pathological stage.

### Validation and Assessment of the Prognostic Signature

In order to validate the validity of the prognosis model, the testing dataset was acquired by a bootstrap method based on resampling of 1000 times (19). The original dataset acted as a training set. In the testing set, we calculated the risk score based on the expression quantity of the model lncRNA and their coefficient according to the training set. Additionally, we compared our prognostic model with existing gene prognostic models by receiver operating curve (ROC) and C-index analysis. The R package of “survival ROC” and the R package of “survcomp” were used to make the ROC curve and calculate the C-index respectively.

### Immune Status and GSEA Analysis

Principal components analysis (PCA) was used to show the different immune statuses of LUAD patients based on the whole gene expression profiles and the prediction model. The “limma R” package and “scatterplot3d R” package were used to complete the PCA analysis. Gene set enrichment analysis (GSEA4.0.3) was used to identify the biological function of the prediction model.

### Statistical Methods

Cox regression analysis, survival analysis, and PCA analysis were achieved in the R software (version 3.6.0). Kaplan–Meier survival analyses were performed by the “survival R” package and “survminer R” package in R software. We verified the prediction model with the “survival R” package, “survminer R” package, “survival ROC R” package, “pheatmap R” package, and the “ggpubr R” package. Gene set enrichment analysis analysis results whose NOM-*P* value < 0.05 was thought to be statistically significant.

## Results

### Establishment of Immune-Related lncRNA

**FIGURE 1:**
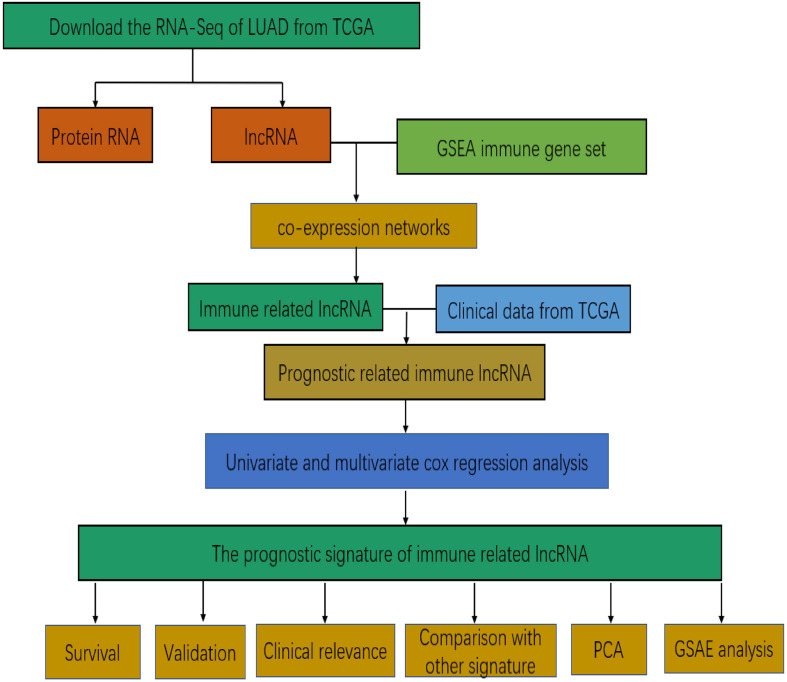
The flow diagram of the whole study base on TCGA database.

**FIGURE 2:**
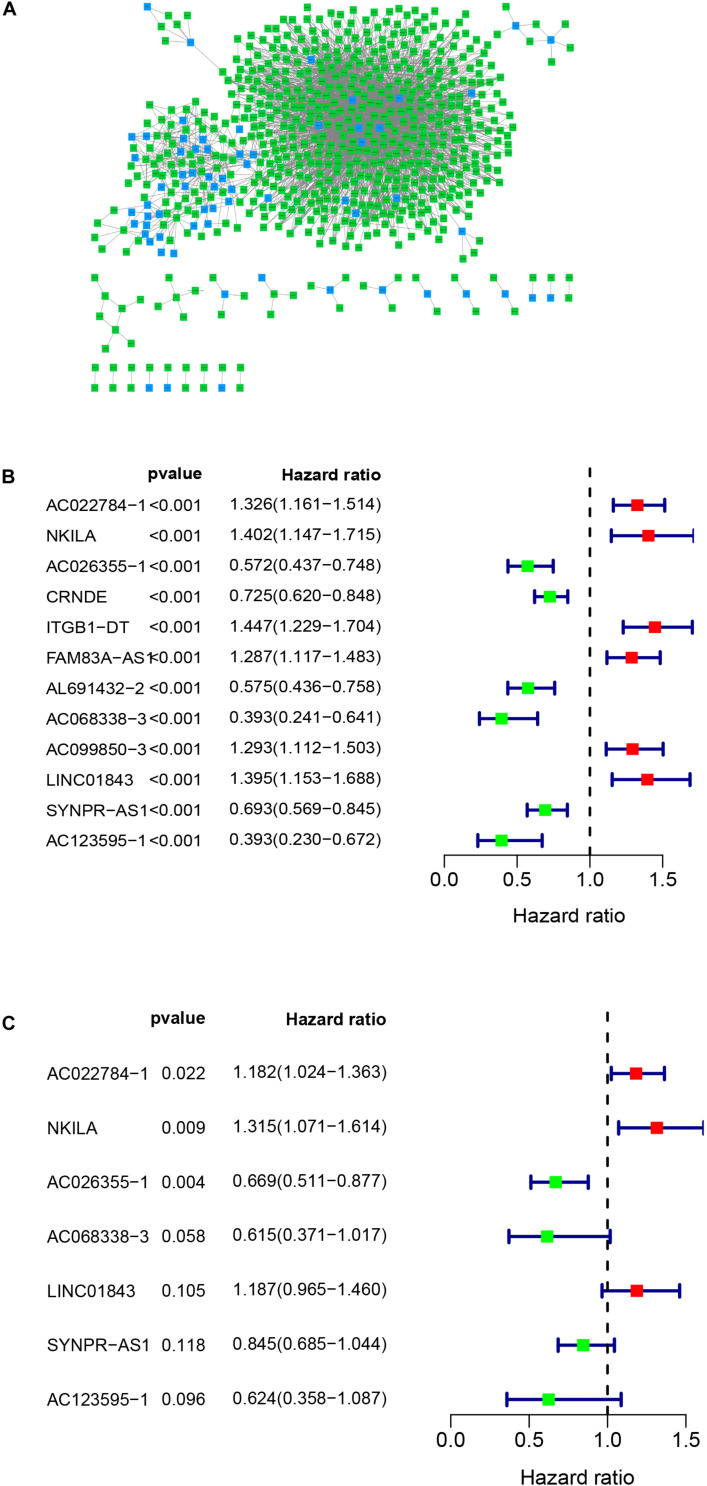
Co-expression network and the result of Cox regression analysis. **(A)** Co-expression network between immune genes and lncRNAs. **(B)** The forest plot of univariate cox regression identified twelve immune-related lncRNAs associated with OS. **(C)** The forest plot of multivariate Cox regression analysis of seven immune-related lncRNAs associated with OS.

Figure 1 presents the flow diagram of this research. A total of 14,144 lncRNAs sequencing data were obtained from TCGA database and 331 immune genes were detected from MSigDB (20). Immune-related lncRNAs were received by building the immune lncRNAs co-expression network through the “limma package” in R studio and Cytoscape3.7.1 (Figure 2A). The co-expression network refers to the relationship between the immune genes and the lncRNAs. Finally, 554 lncRNAs were identified (*P* ≤ 0.001).

### Construction and Validation of the Immune-Related lncRNA Signature of LUAD

**FIGURE 3:**
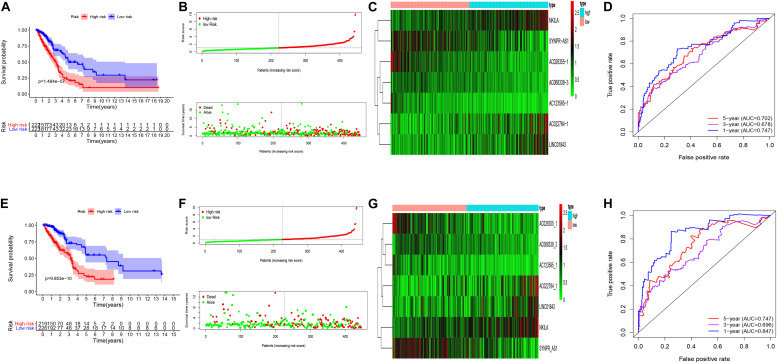
Kaplan–Meier curve survival analysis, risk score analysis, heatmap, Time–ROC curve analysis. **(A)** Kaplan–Meier curve, **(B)** risk score, **(C)** heatmap, **(D)** time–ROC curve of the immune-related lncRNA signature in the training cohort. **(E)** Kaplan–Meier curve, **(F)** risk score, **(G)** heatmap, and **(H)** time–ROC curve of the immune-related lncRNA signature in the testing cohort.

**FIGURE 4:**
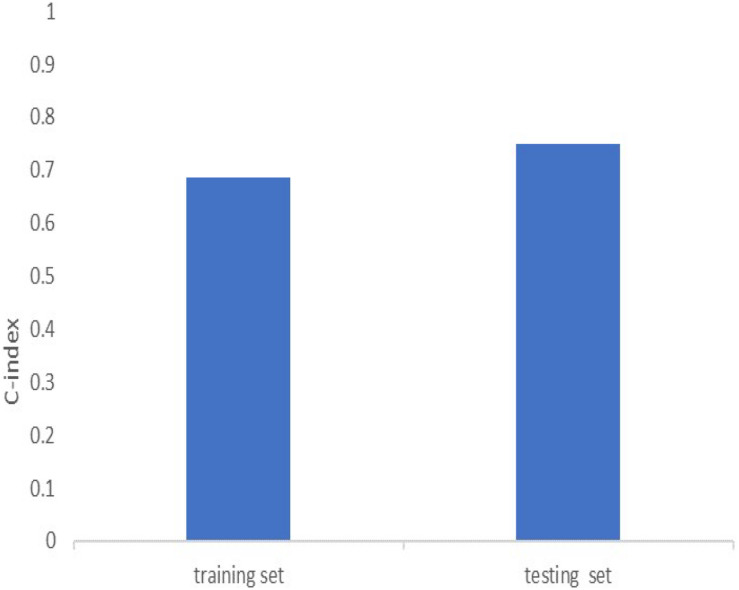
C-index of the immune-related lncRNA signature in the training cohort and the testing cohort.

Univariate cox regression analysis was first applied to recognize the predictive model. A total of 12 immune-related lncRNAs had a conspicuous connection with the overall survival rate (*P* < 0.001). The forest plot of the univariate cox regression is shown in Figure 2B. Then, 12 immune-related lncRNAs were selected and put into the multivariate Cox regression analysis. The result revealed that seven immune-related lncRNAs can act as independent prognostic factors for LUAD. The forest plot of the multivariate cox regression is shown in Figure 2C. Finally, the seven immune-related lncRNAs were selected as our model to predict LUAD’s prognosis (Table 1). The risk score of each patient was computed according to the following formula. Risk score = 0.167 × expression quantity of AC022784-1 + 0.274 × expression quantity of NKILA + (−0.401) × AC026355-1 + (−0.487) × expression quantity of AC068338-3 + 0.171 × expression quantity of LINC01843 + (−0.168) × expression quantity of SYNPR-AS1 + (−0.472) × expression quantity of AC123595-1. We obtained a high-risk group (*n* = 222) and a low-risk group (*n* = 223) according to the median risk scores. The overall survival (OS) of patients in the high-risk group are shorter than in the other group (*P*-value = 1.484e-7; Figure 3A). In our survival analysis, the survival rate of the LUAD patients in the low-risk group was 66% after 3 years, 46% after 6 years, however, the survival rate in the high-risk group was only 48% after 3 years, 17% after 6 years, respectively. The risk score curve and survival status data of these two different groups are shown in Figure 3B. The abscissa axis of the risk score curve and survival status data were ranked by the risk score value. This result showed that the mortality of LUAD patients in the high-risk group was much higher than patients in the other group. To show the expression difference of our model lncRNAs, we used a heat-map plot (Figure 3C). The heat-map showed that the expression of lncRNAs (NKILA, AC022784-1, LINC01843) were obviously up-regulated in patients in the high-risk group, whereas the model lncRNAs (SYNPR-AS1, AC026355-1, AC068338-3, AC123595-1) were down-regulated. In the low-risk group, the expression of NKILA, AC022784-1, and LINC01843 were correspondingly decreased. The accuracy of the prognostic model was shown in the ROC curve. The area under the curve (AUC) values of the ROC curve of the 1-, 3-, and 5-year OS were 0.747, 0.678, and 0.702, respectively (Figure 3D). Then, the prognostic model was verified in the testing dataset, the OS in the high-risk group was significantly worse than that in the low-risk group (*P*-value = 9.853e-10; Figure 3E). The risk score curve and survival status data of the two different groups in the testing dataset are shown in Figure 3F. The heat-map plot of the testing dataset is shown in Figure 3G. Similarly, the AUC values of the 1-, 3-, and 5-year OS in the testing dataset were 0.847, 0.696, and 0.747, respectively (Figure 3H). The C-index for OS predictions in the training dataset and testing dataset were 0.687 (95% CI, 0.639–0.735) and 0.749 (95% CI, 0.707–0.791) (Figure 4).

**TABLE 1:** The seven immune-related lncRNAs detected from multivariable Cox regression analysis.

**ID**	**Coef**	**HR**	**HR.95L**	**HR.95H**	***P* value**
AC022784-1	0.167	1.182	1.024	1.363	0.022
NKILA	0.274	1.315	1.071	1.614	0.009
AC026355-1	–0.401	0.669	0.511	0.877	0.004
AC068338-3	–0.487	0.615	0.371	1.017	0.058
LINC01843	0.171	1.187	0.965	1.460	0.105
SYNPR-AS1	–0.168	0.845	0.685	1.044	0.118
AC123595-1	–0.472	0.624	0.358	1.087	0.096

### Independent Analysis Between the Prognostic Model and the Other Clinical Variables

**FIGURE 5:**
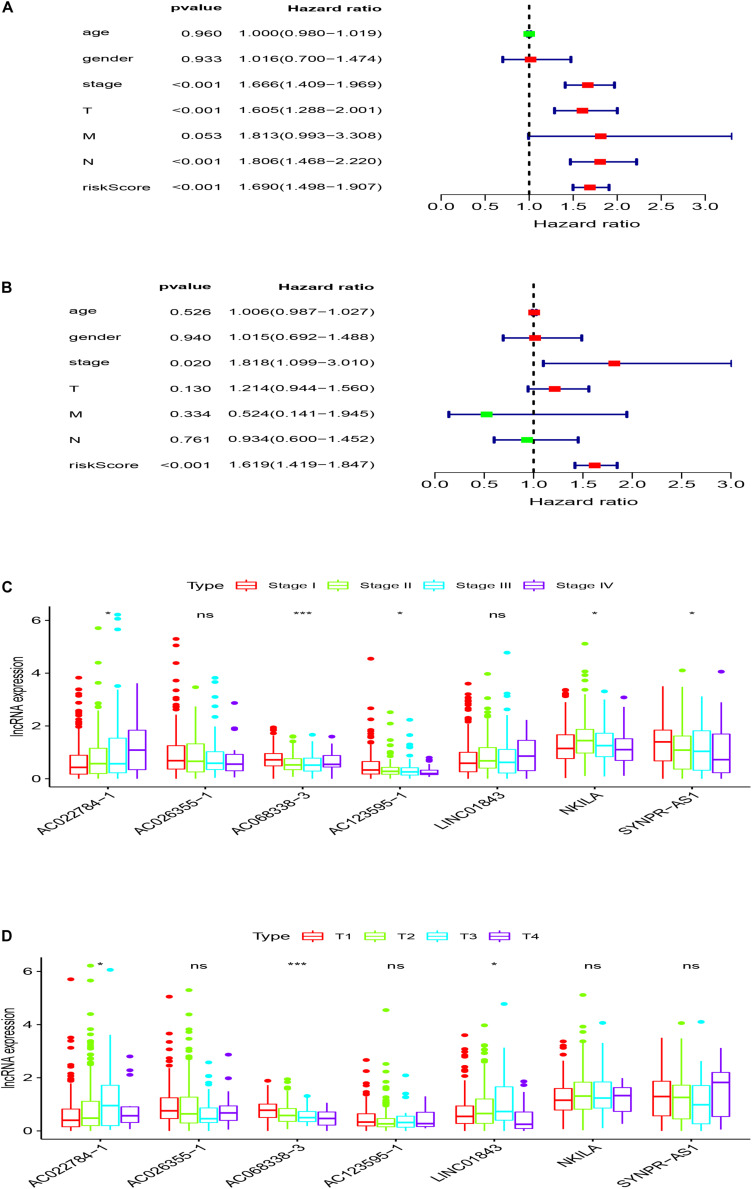
Stratification analyses of clinicopathological characteristics. **(A)** Univariate independent prognostic analysis forest map of the prognostic model and LUAD clinicopathological characteristics. **(B)** Multivariate independent prognostic analysis forest map of prognostic model and LUAD clinicopathological characteristics. **(C)** Stratification analyses of all patients adjusted to stage using the signature of seven immune-related lncRNAs. **(D)** Stratification analyses of all patients adjusted to T stage using the signature of seven immune-related lncRNAs.

In order to evaluate whether the survival prognosis of these lncRNAs were independent of other clinical factors. We used cox regression analysis to analysis LUAD clinical characteristics based on the predictive model. Univariate independent prognostic analysis illustrated that the risk score, stage, pathological T stage, and pathological N stage have statistical difference when connected with overall survival (*P* < 0.001) (Figure 5A). Multivariate independent prognostic analysis illustrated that stage and risk score can be considered as independent predicted factors of LUAD (*P* < 0.05) (Figure 5B). In conclusion, univariate and multivariate independent prognostic analysis illustrated that the prediction model was an independent predicted factor (*P* < 0.05) (Table 2). Furthermore, we used stratification analysis to identify the independence of the prediction model (Figures 5C,D). For stage, AC068338-3 was significantly up-regulated in early stage LUAD (*P* < 0.001). AC022784-1 was determined to be over expressed in patients of later stage LUAD (*P* < 0.05). SYNPR-AS1 was significantly up-regulated in early stage LUAD (*P* < 0.05). AC068338-3 had obviously statistical significance comparing with the pathological T stage (*P* < 0.001). The expression of AC068338-3 was down-regulated with the development of the pathological T stage.

**TABLE 2:** Univariate and multivariate Cox regression analyses among other clinical factors.

**Variables**	**Univariate model**				**Multivariate model**			
	**HR**	**HR.95L**	**HR.95H**	***P* value**	**HR**	**HR.95L**	**HR.95H**	***P* value**
Age	1.000	0.980	1.019	0.960	1.006	0.987	1.027	0.526
Gender	1.016	0.700	1.474	0.933	1.015	0.692	1.488	0.940
Stage	1.666	1.409	1.969	0.000	1.818	1.099	3.010	0.020
T	1.605	1.288	2.001	0.000	1.214	0.944	1.560	0.130
M	1.813	0.993	3.308	0.053	0.524	0.141	1.945	0.334
N	1.806	1.468	2.220	0.000	0.934	0.600	1.452	0.761
RiskScore	1.690	1.498	1.907	0.000	1.619	1.419	1.847	0.000

#### Comparison With Other Existing Prognostic Signatures

**FIGURE 6:**
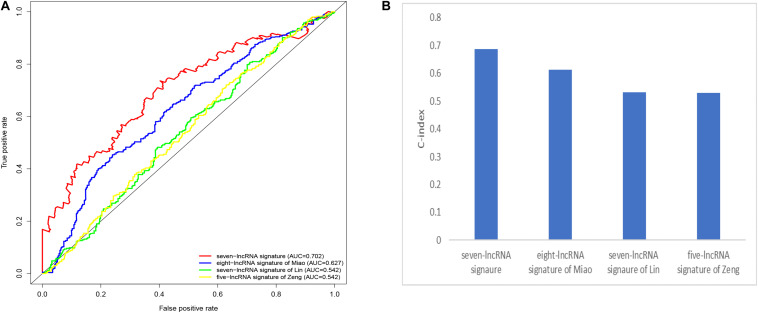
Comparison of the receiver operating characteristic (ROC) curve **(A)** and c-index **(B)** for different prognostic signatures.

We compared our prognostic model with other published prognostic signatures (21–23). In Figure 6A, the AUC of ROC for 5-year OS in our seven immune-related lncRNA model was 0.702. The C-index is 0.687. Comparing with other prognostic models, our model has superior predictive sensitivity and specificity. The AUC of the eight-lncRNA signature of Miao, seven-lncRNA signature of Lin, and five lncRNA signature of Zeng are 0.627, 0.542, and 0.542, respectively. The C-index of the eight-lncRNA signature of Miao, seven-lncRNA signature of Lin, and five lncRNA signature of Zeng are 0.613, 0.531, and 0.530, respectively (Figure 6B).

#### The Immune State of Different Risk Groups and Functional Enrichment Analysis

**FIGURE 7:**
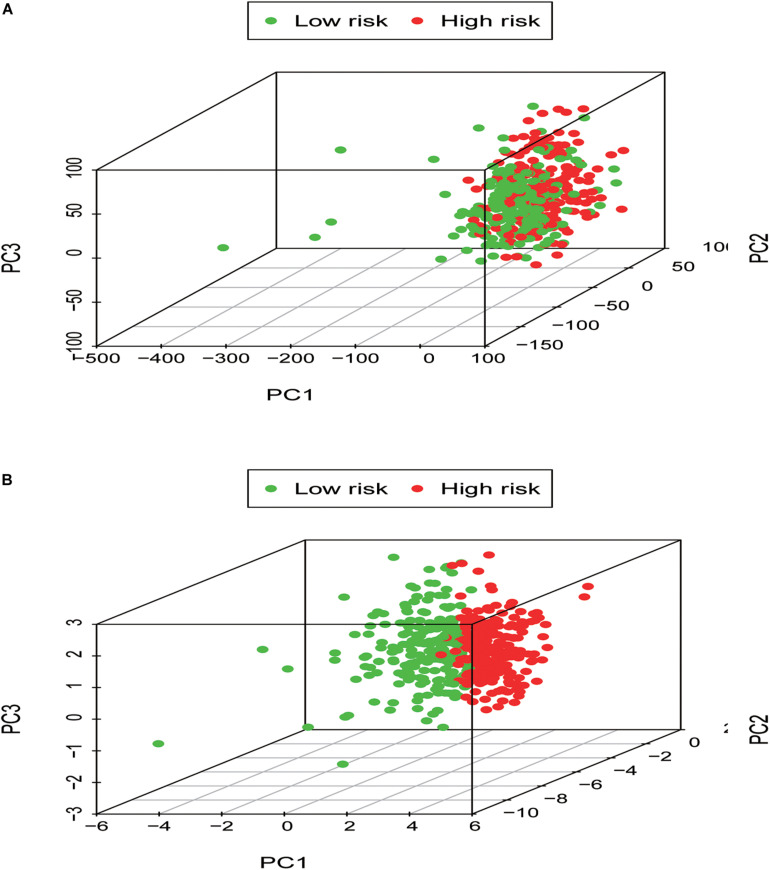
Low- and high-risk groups displayed different immune status. **(A)** Principal components analysis between low- and high-risk groups based on whole gene expression profiles. **(B)** Principal components analysis between low- and high-risk groups based on the signature of seven immune-related lncRNAs.

**FIGURE 8:**
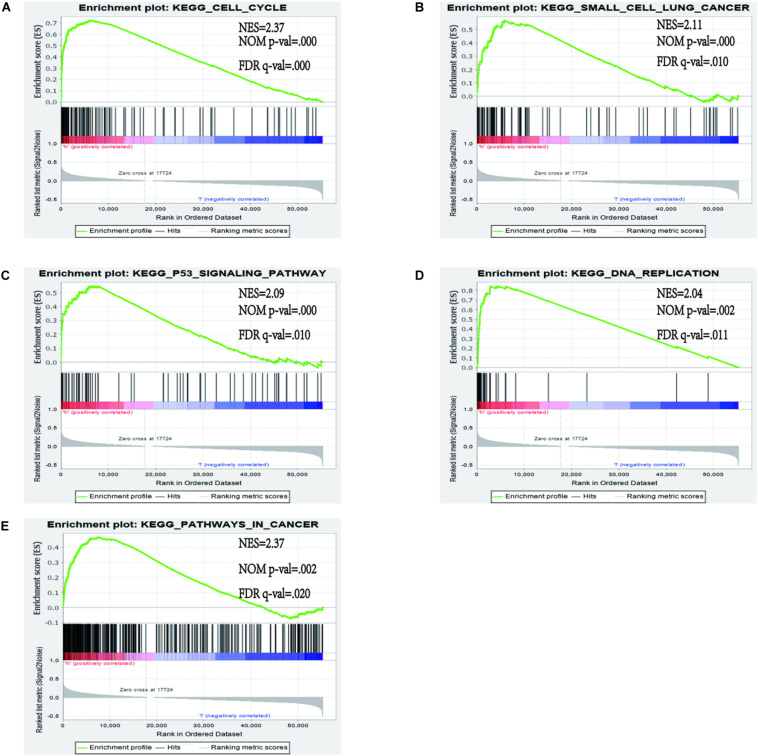
Gene set enrichment analysis (GSEA) between high and low immune risk groups. **(A)** CELL_CYCLE; **(B)** SMALL_CELL_LUNG_CANCER; **(C)** P53_SIGNALING_PATHWAY; **(D)** DNA_REPLICATION; **(E)** PATHWAYS_IN_CANCER.

In order to identify the discrepancy between the two risk groups based on our model lncRNAs and the total gene expression, PCA analysis was applied to our progression (Figure 7). The result illustrated that patients in these two groups were spread in different directions. However, the model lncRNAs divided LUAD patients into two specific sections, showing that the immune status of LUAD patients were quite different in the two groups. To identify the unknown function of the seven-lncRNAs model, we used GSEA analysis to find possible biological functions of the seven-lncRNA model of LUAD (Figure 8). The GSEA analysis showed that five tumor gene sets (“P53_SIGNALING_PATHWAY,” “DNA_REPLICATION,” “CELL_CYCLE,” “SMALL_CELL_LUNG_CANCER,” “PATHWAYS_IN_CANCER”) were obviously enriched in the high-risk group. The result indicated that these associated biological pathways can significantly affect the tumorigenesis of LUAD.

## Discussion

Lung adenocarcinoma is the most common pathological subtype and it has been widely detected over the years via unique genetic changes and various prognostic factors (24–26). Due to unknown pathogenesis, the mortality of LUAD patients is still high and the treatment outcome is also unsatisfactory (27). In recent years, lncRNAs have played a more significant role in the development of tumors and the disorder of lncRNA may be considered as a crucial factor in the activities of tumors (28, 29). We have found that lncRNA participated in 33 kinds of cancers. Moreover, the importance of immune-related genes in tumor progression and immunotherapies has become apparent (30–32). Immune-related lncRNA helps to prioritize cancer-related lncRNA and distinguish cancer subtypes based on specific immunological characteristics. In addition, in recent years, prognostic biomarkers combined with the tumor immune microenvironment have already emerged (33–36). The value of immune-related lncRNA has been shown in many cancers such as hepatocellular carcinoma (HCC), anaplastic glioma (37), glioblastoma multiforme (38), diffuse large B cell lymphoma (39), and breast cancer. In the research of HCC, Zhang et al. identified that the immune-related lncRNA signature not only had a significant effect on survival prognosis but also had the possible role of evaluating the response to ICB (immune checkpoint blockade) immunotherapy (40). Shen et al. discovered the 11-lncRNA signature of breast cancer and analyzed the correlation between the lncRNA prognostic signature and the infiltration of immune cell subtypes (41). This novel field can improve patient treatment in the era of immunotherapy. Although the preciseness of survival prediction has still not reached its potential and many limitations remain. Therefore, it is necessary to identify accurate markers and to choose suitable immune therapies to enhance the survival rate of LUAD.

Recently, several studies have revealed prognostic markers of the tumor immune microenvironment that are capable of predicting the prognosis of the tumor. Shen et al. found that an immune gene model can act as a possible marker to predict the prognosis of clear renal clear cell cancer (42). Yang et al. revealed that a prediction immune model can forecast the survival outcome of cervical cancer (43). Chen et al. reflected that a nine immune gene model has prognostic value for HCC (44). Nowadays, prognostic biomarkers related to tumor immunity in lung cancer are still lacking. In our research, we tried to detect the prognostic model of LUAD based on immune-related lncRNA.

In our study, we demonstrated that a seven immune-related lncRNA can improve prognosis prediction in LUAD. Gene set enrichment analysis analysis revealed that these lncRNAs were enriched in the pathways of “P53_SIGNALING_PATHWAY,” “DNA_REPLICATION,” “CELL_CYCLE,” “SMALL_CELL_LUNG_CANCER,” and “PATHWAYS_IN_CANCER,” A recent study illustrated that an immune regulatory protein can induce apoptosis of lung carcinoma cells through the P53 signaling pathway (45). The P53 signature pathway is closely connected to the progression of lung carcinoma. César Muñoz-Fontela et al. indicated that the p53 signaling pathway has an extensive impact on immune responses (46). For example, it can regulate the immune signal to participate in the immune reaction and effect autoimmunity by restraining inappropriate response of inflammatory cytokines (47–49). DNA replication is a significant molecular mechanism of tumorigenesis. Macheret et al. indicated that the enhancement of DNA replication is an important symbol of cancer progression (50). A recent study revealed that ailanthone can suppress NSCLC growth by restraining DNA replication through reducing RPA1 (replication protein A1) (51). In our research, the prediction signature was not only connected with the immune response but also with tumorigenesis. Compared with previous studies, our study first constructed the prognostic model based on immune-related lncRNAs.

In this study, the immune-related lncRNAs prediction model was established by univariate and multivariate Cox regression analysis. In order to verify the efficiency of our model, we used a K–M survival curve to illustrate the survival time of the two groups. The *P* value of the K–M survival curve was 1.484e-7, which indicated that our predicated model had a strong correlation with the survival outcomes of LUAD patients. Furthermore, we validated our prognostic signature in the testing set. The AUC of ROC of 5 years were 0.702 and 0.747, respectively in the training dataset and the testing dataset, which showed that this model had superior accuracy. Our prognostic model was also more superior than other prognostic signatures by comparison. Principal components analysis indicated that the prediction model was equipped to separate LUAD patients into different groups according to their immune status. Finally, we used GSEA analysis to detect the biological functions of our prediction model. The result powerfully identified that these lncRNAs participated in the progression of the tumors.

## Conclusion

In conclusion, in this study, we constructed an immune-related lncRNAs model of LUAD. The finding illustrated that the seven immune-related lncRNAs prognosis model was efficient in predicting the clinical prognosis. Furthermore, studies based on the immune response and lncRNA not only enhanced the diagnosis rate but also gave us a new direction for immune therapy.

## Data Availability Statement

RNAseq FPKM data was downloaded from the TCGA GDC data portal (https://portal.gdc.cancer.gov/) under the project ID of TCGA-LUAD. Immune genes were downloaded from The Molecular Signatures Database (https://www.gsea-msigdb.org/gsea/msigdb/) under the accession numbers of Immune system process M13664 and Immune response M19817.

## Author Contributions

Y-QQ and J-PL designed the study. RL and XL collected the literature. CH, T-TL, and JY analyzed the data. J-PL drafted the manuscript. Y-QQ modified the manuscript. All authors read and approved the final manuscript.

## Conflict of Interest

The authors declare that the research was conducted in the absence of any commercial or financial relationships that could be construed as a potential conflict of interest.

## Footnotes

1 https://www.cancer.gov/about-nci/organization/ccg/research/structural-genomics/tcga

2 https://www.gsea-msigdb.org/gsea/msigdb

## Abbreviations

LUAD: lung adenocarcinoma
TCGA: The Cancer Genome Atlas
MSigDB: Molecular Signatures Database
SCLC: small cell lung cancer
NSCLC: non-small cell lung cancer
OS: overall survival
ROC: receiver operating curve
AUC: area under ROC curve
PCA: principal components analysis
GSEA: gene set enrichment analysis.
